# Fused-Filament Fabrication of Short Carbon Fiber-Reinforced Polyamide: Parameter Optimization for Improved Performance under Uniaxial Tensile Loading

**DOI:** 10.3390/polym14071292

**Published:** 2022-03-23

**Authors:** Carlos Belei, Jana Joeressen, Sergio T. Amancio-Filho

**Affiliations:** BMK Endowed Professorship for Aviation, Institute of Materials Science, Joining and Forming, Graz University of Technology—TU Graz, Kopernikusgasse 24/1, 8010 Graz, Austria; carlos.belei@tugraz.at (C.B.); jana@joeressen.net (J.J.)

**Keywords:** additive manufacturing, fused filament fabrication, thermoplastic-based composites, short fiber-reinforced polyamide

## Abstract

This study intends to contribute to the state of the art of Fused-Filament Fabrication (FFF) of short-fiber-reinforced polyamides by optimizing process parameters to improve the performance of printed parts under uniaxial tensile loading. This was performed using two different approaches: a more traditional 2k full factorial design of experiments (DoE) and multiple polynomial regression using an algorithm implementing machine learning (ML) principles such as train-test split and cross-validation. Evaluated parameters included extrusion and printing bed temperatures, layer height and printing speed. It was concluded that when exposed to new observations, the ML-based model predicted the response with higher accuracy. However, the DoE fared slightly better at predicting observations where higher response values were expected, including the optimal solution, which reached an UTS of 117.1 ± 5.7 MPa. Moreover, there was an important correlation between process parameters and the response. Layer height and printing bed temperatures were considered the most influential parameters, while extrusion temperature and printing speed had a lower influence on the outcome. The general influence of parameters on the response was correlated with the degree of interlayer cohesion, which in turn affected the mechanical performance of the 3D-printed specimens.

## 1. Introduction

Additive manufacturing (AM) processes are highly valued nowadays due to their ability to produce parts with a high degree of complexity. Such a characteristic is relevant in a context where there is a constant search for alternatives that could reduce the required raw material, as well as the number of manufacturing steps. By fulfilling these requirements, AM processes yield considerable resource savings that begin during the production of a component.

Apart from the production, it is also important that its implementation into a given system or structure results in an overall gain in efficiency, which in turn would readily translate into lower resource consumption. Over the course of the last decades, a widely adopted strategy to address this challenge is to decrease the weight of produced components. This is achieved by clever geometric design or by using materials with high strength-to-weight ratios, which offer the desired weight reduction without compromising the mechanical performance. For this second approach, materials such as lightweight metallic alloys and fiber-reinforced composites are useful.

In comparison to lightweight alloys, the processing of fiber-reinforced polymer composites is generally easier due to the lower energy involved and their low reactivity to the surrounding environment. This, coupled with competitive strength-to-weight ratios (which in many cases is even superior in comparison to some lightweight alloys [1]), make polymeric composites a class of material that is highly sought after for many applications [2,3,4,5,6]. Even though the market for composites is still dominated by thermoset-based ones [7,8], the use of thermoplastic matrices is trending upward, bringing along advantages such as the much longer shelf life, absence of curing stages, higher impact toughness and recyclability [6,9,10,11]. Moreover, thermoplastic-based composites are also being increasingly deployed in the production of additively manufactured components [12,13,14], which by itself entails key benefits, as already mentioned.

For the AM of thermoplastic composites, one process stands out as the most popular: the fused filament fabrication (FFF). The general idea is controlled extrusion of a thermoplastic filament through a heated nozzle. After reaching a specified processing temperature, the heated thermoplastic polymer is deposited onto a platform. The relative movement of the nozzle with respect to the platform defines the shape of the deposited layer. The process proceeds by stacking several layers on top of each other until the solid part is finished.

The manufactured (commonly referred to as 3D-printed) part can be reinforced either by continuous or short fibers. Printing continuous fiber reinforced parts by FFF is more difficult due to the complex nature of the apparatus necessary to embed the fibers in a thermoplastic matrix [15,16]. Currently, a few approaches have been proposed to achieve such a task [16,17,18,19,20], most remarkably the one patented by Markforged Inc. (Watertown, MA, USA) [21,22]. By contrast, although considerably inferior in terms of mechanical properties [15,23,24,25,26], short-fiber reinforced (SFR) thermoplastics do not require any extra feeding, fiber impregnation and/or cutting mechanisms to be printed. This enables virtually any commercial FFF machine to perform the task with only minor modifications (such as wider, hardened nozzles to avoid premature wear [27,28] and blockage [29]). This relative simplicity in terms of processing requirements turns these materials into a viable alternative to further increase the mechanical performance of thermoplastic polymers manufactured by FFF, without compromising their light weight [15,30].

Among the most used thermoplastic matrices for SFR filaments, polyamide-based polymers are of special interest, since they provide a good trade-off between mechanical performance and processability. Many authors dedicated their efforts to explore the FFF of polyamide-based SFR filaments under a number of different aspects. Liao et al. [31] concluded that adding 10% of short carbon fibers to a PA12 matrix improved tensile and flexural strength of printed parts without compromising their impact resistance. Badini et al. [32] demonstrated the dependence of fiber orientation on the anisotropy of SFR PA6 specimens, more so than matrix-related factors, such as crystallinity or porosity. Pei et al. [33] optimized the process with respect to the void content for a PA filament reinforced with 20% short carbon fibers, thereby finding an important correlation between process parameters and void volume fraction. Naranjo-Lozada et al. [23] observed small improvements in tensile strength on specimens printed with chopped carbon fiber reinforced PA6 compared to unreinforced ones. Peng et al. [34] observed that the printing bed temperature had a positive influence on the tensile strength of specimens printed with a tailor-made PA6 filament reinforced with 10% short carbon fibers. Handwerker et al. [35] investigated the effect of annealing conditions on the mechanical properties of chopped carbon fiber-reinforced PA6 parts along the build-up direction. The authors concluded that after 6 h of heat treatment at 200 °C, 3D-printed parts increased their elastic modulus threefold with an increase in ultimate tensile strength of more than 50%. Dul, Fambri and Pegoretti [36] compared 3D-printed PA/CF parts with compression-molded PA/CF ones based on their mechanical and functional performances. The authors concluded that, while there was a decrease in elastic modulus compared to the compression-molded specimens, the 3D-printed ones had a higher electrical resistivity. De Toro et al. [37] compared short-carbon fiber reinforced PA6 parts produced by FFF and injection molding. The authors concluded that the difference in tensile strength of up to 21% in favor of injected parts was not translated to the compression test results, where the performance of FFF and injected parts was similar. Abderrafai et al. [38] studied the 3D-printing of a PA12 matrix with different types of short carbon fibers (chopped and milled) and observed important differences in mechanical performance. Ferreira et al. [39] studied the machinability of SFR PA12 parts manufactured by FFF, concluding that machining of such parts is indeed possible without layer delamination.

Despite the large content covered by the cited studies and others, there is still a considerable field of unexplored aspects. For one, none of the aforementioned publications conducted a comprehensive process optimization involving the influence of several key FFF parameters at once, adopting a mechanical property of the printed SFR polyamide as a response. Such a study could contribute to the state of the art of 3D-printed SFR polyamides by revealing possible parameter combinations for optimal mechanical performance, as well as aspects such as parameter interactions and parameter–microstructure–property relationships. Therefore, we aimed to perform such an optimization study as a means to improve the performance of printed SFR polyamide parts under uniaxial tensile loading. In order to evaluate possible differences in terms of prediction power, two different models were constructed: one based on the more traditional 2k full factorial DoE and another based on a multiple polynomial regression using an algorithm implementing machine learning principles such as train–test split and cross-validation. Resulting prediction models from both approaches were then compared to each other in terms of accuracy and bias. Finally, the results from both models were related to microstructure and temperature evolution during the process. Apart from providing optimal, exact parameter windows and values, this work intends to contribute as a reference that can be applied even to situations where materials and 3D-printer models are different to the ones used herein.

## 2. Materials and Methods

### 2.1. Filament Material

The specimens used in this study were produced using a BASF Ultrafuse PAHT CF15 filament (Emmen, The Netherlands), with a diameter of 2.85 mm. This filament consists of a commercial-grade polyamide matrix reinforced with short carbon fibers at a volume fraction of 6.5 ± 0.2%. According to the supplier’s material technical specifications [40], the glass transition and melting temperatures of the filament are 70 °C and 234 °C, respectively. During the FFF process, the filament spool was stored in an enclosed box with an internal heating unit, which was responsible for keeping the filament at a constant temperature of 40 °C. To keep moisture absorption by the polymer at a reduced level, packages of silica gel were placed into the enclosed box. The determination of absorbed water content in the PA-CF filament was not carried in this work, as the authors intended to evaluate the FFF printability of the filament in its as-delivered condition.

### 2.2. Process Setup, Specimen Geometry and Mechanical Testing

The FFF process was carried out using an Ultimaker S5 3D-printer (Utrecht, The Netherlands). A printing bed consisting of a 4.0 mm thick glass plate was used during the study, without tape or adhesive. The printing nozzle had a diameter of 0.6 mm; its tip was protected with a ruby cone to avoid premature wear due to the abrasive nature of the filament.

**Figure 1:**
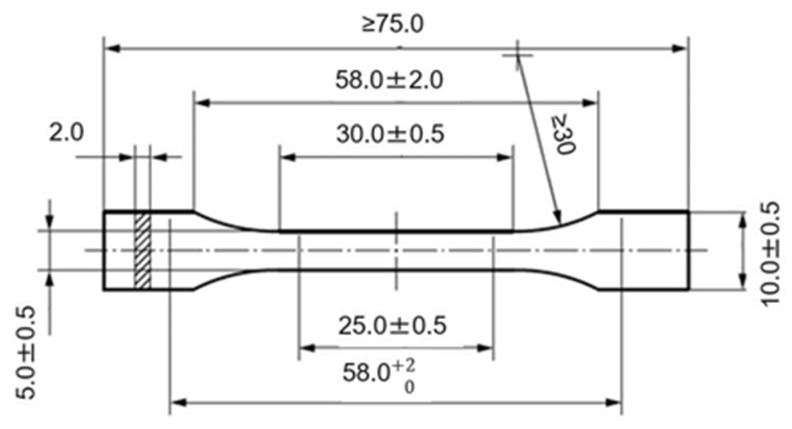
Specimen dimensions (in mm) based on ISO 527 I-BA.

**Figure 2:**
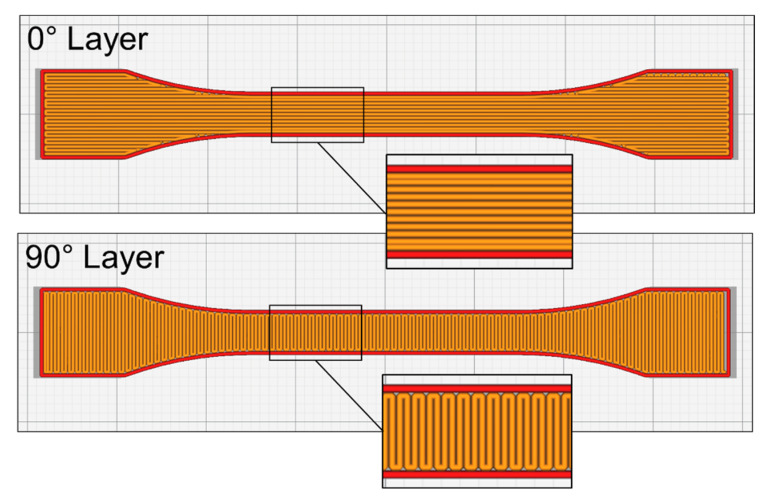
Infill pattern of successive layers within a given tensile specimen.

The dimensions of the 3D-printed specimens were based on the ISO 527 I-BA standard, as presented in Figure 1. Slicing was carried out using the Ultimaker Cura 4.11.0 slicing software (Utrecht, The Netherlands), whereby all process parameters for each specimen were defined. The orientation of the deposited roads with respect to the specimen length followed a 0°/90° sequence (Figure 2). After printing, the heating unit of the printing bed was switched off, and the specimens were left to cool down on the bed until it reached a temperature of 70 °C.

Mechanical testing was performed on a ZwickRoell Z100 test machine (Ulm, Germany) equipped with a 100 kN load cell. Specimens were fixed using non-positive clamping with self-gripping wedge grips. The initial distance between the grips was 50 mm. Tests were conducted with a speed of 2 mm/min at a temperature of 19 °C. No extensometer was used. The registered force was converted to stress using only the initial cross-sectional area of each specimen.

### 2.3. Process Parameters and Data Analysis

The evaluated process parameters and their respective ranges are listed in Table 1. The ranges of extrusion and printing bed temperatures, as well as printing speed and layer height were selected based on the material datasheet provided by the filament supplier. All the other process parameters remained constant throughout the study.

#### 2.3.1. Prediction Model Based on 2k Full Factorial DoE

**Figure 3:**
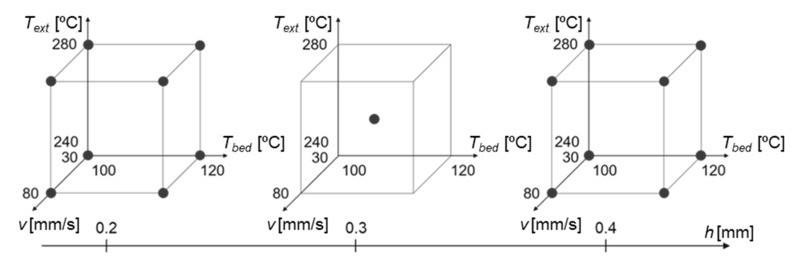
Black points indicate the conditions evaluated for the 2k full factorial DoE.

Within the selected ranges, a 2k full factorial design of experiments with a center point was deployed (Figure 3), adopting the ultimate tensile strength (UTS) of the tested specimens as the response. Four replicas of each condition were initially printed and tested. Based on the test results, a prediction model was generated using Minitab 19 (State College, PA, USA). The statistical significance of model features was assessed using analysis of variance (ANOVA) with α = 0.05. Non-informative features present in the model were removed by backward elimination, with α = 0.10. An optimized set of parameters was obtained by maximizing the regression equation within the tested intervals.

**Figure 4:**
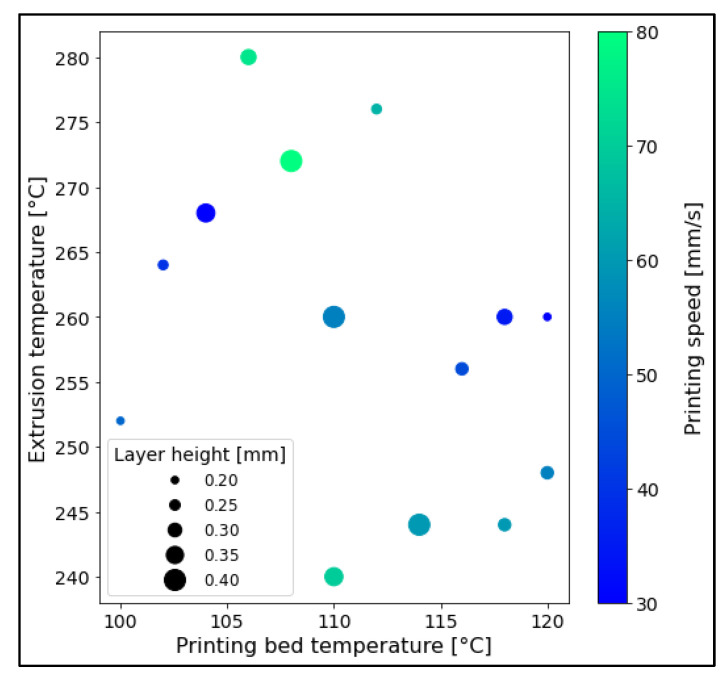
Conditions evaluated for the validation of the DoE-based prediction model.

In order to validate the prediction model as a whole, 15 parameter combinations randomly scattered across the evaluated ranges were used to produce four specimens each (Figure 4). Moreover, eight replicas 3D-printed specifically with the predicted optimized set of parameters were tested and their results compared to the predicted optimal response.

#### 2.3.2. Prediction Model Based on Polynomial Regression Using Machine Learning Principles

While creating a prediction model based on the 2k full factorial DoE, a multiple polynomial regression using an algorithm based on machine learning (ML) principles was implemented to compute an additional model. With this, a performance comparison between both methods (DoE and ML-based) could be established. In the present case, the datasets used for calculating and for validating the prediction model as described in Section 2.3.1 were merged into a single, complete dataset with 32 unique parameter combinations and a total of 166 data points.

The operations described here were executed with functions present in the scikit-learn 0.22 library available for Python. The complete dataset was split into training and test sets in a proportion of 80:20. Training and test data were standardized separately using the MinMax scaler, a built-in function that transformed each parameter value of a given data point (*x_i_*) based on minimum and maximum values for each respective parameter within a given dataset (*x_max_* and *x_min_*, respectively), as shown in Equation (1). To avoid data leakage, this transformation was performed based on the minima and maxima from the training set only.

(1) $$x_{std}=\left(x_{i}-x_{min}\right)/\left(x_{max}-x_{min}\right)\;$$

**Figure 5:**
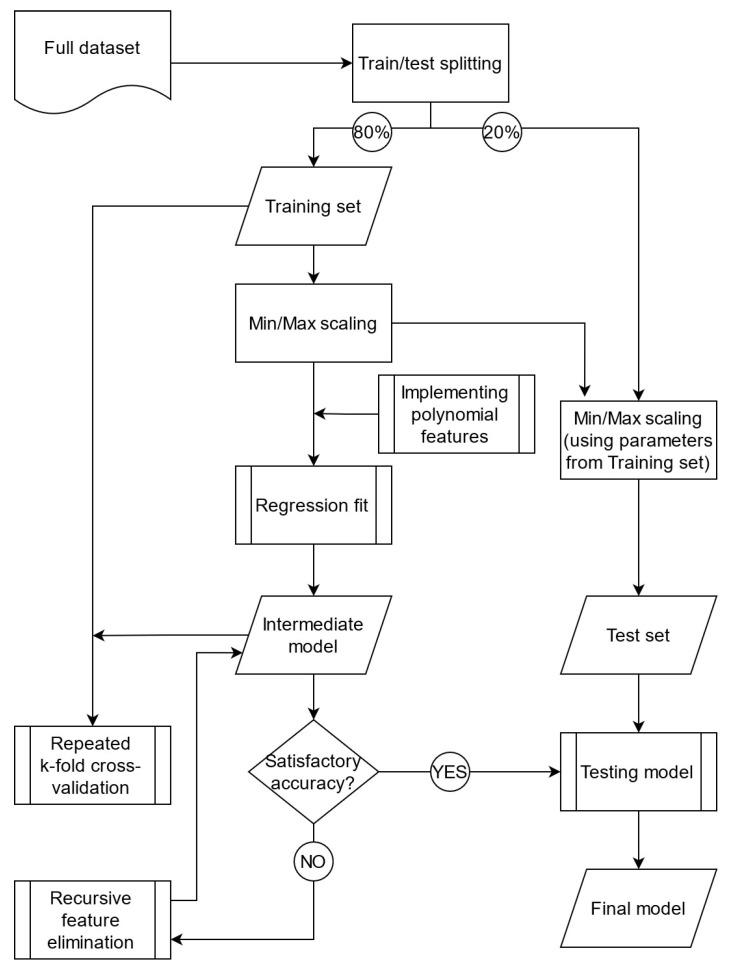
ML-based algorithm for the multiple polynomial regression applied on the complete experimental dataset.

Then, the algorithm fitted a multiple polynomial regression of degree 2 on the training set, followed by recursive feature elimination (RFE), a method applied to decrease the model complexity by removing features based on their influence on the overall response [41,42]. For each loop of RFE, the skill of the model was scored based on the mean accuracy of a 5-time repeated 5-fold cross-validation (CV) performed on the training set. The feature elimination loop stopped when the model accuracy according to the CV score reached its maximum. The test set was held out after the initial splitting, being utilized only for the model evaluation based on its final metrics (i.e., RMSE and R^2^). An overview of the implemented algorithm is presented by Figure 5.

### 2.4. Temperature Measurements

**Figure 6:**
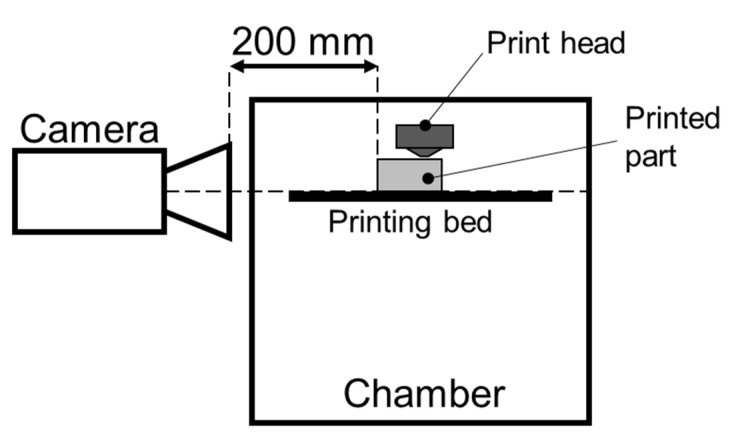
Schematics of the infrared camera placement with respect to the printed part.

In order to measure the temperature evolution during and after the process, an InfraTec Variocam HD (Dresden, Germany) was used. The camera was positioned outside the printer chamber at a distance of 0.2 m from the specimen at an incidence angle of 0° (see Figure 6). An emissivity factor of 0.95 was set based on a local calibration, which was carried out by comparing the temperature results given by the infrared camera with the readings from a Type-K thermocouple attached to a part being printed (*T_ext_* = 280 °C and *T_bed_* = 120 °C). The emissivity factor found by this method is in good agreement with the literature [43,44]. The exposed surface of the printing bed (i.e., the surface not being printed on) was covered with a dark, opaque tape in order to reduce reflection. To evaluate the overall tendencies in terms of temperature gradients in greater detail, specimens for temperature measurements were printed with a thickness of 5 mm.

### 2.5. Microstructural Analysis and Fractography

Microstructural analysis prior and after mechanical testing was carried out using scanning electron microscopy (SEM) with a Tescan Mira-3 machine (Brno, Czech Republic). Prior to being analyzed, the specimens were carbon sputtered. The sputtered surfaces were observed using the secondary electron detector with an acceleration voltage of 5 kV, at a working distance of 50 mm and a chamber pressure of 10^−1^ Pa.

## 3. Results and Discussion

### 3.1. Process Optimization

#### 3.1.1. Prediction Model Based on the 2k Full Factorial DoE

The average UTS results for each parameter combination in the original dataset (i.e., the one comprising the 2k full factorial DoE, see Figure 3) are presented in Table 2. The prediction model calculated based on this data is represented by Equation (2). Despite the parameter values being within the ranges recommended by the filament supplier, the UTS values varied considerably from one condition to another. According to the adjusted R^2^, 79.5% of the observed variance in the experimental data could be explained by the proposed model (see model summary, Table 3), which can be considered reasonable.

(2) $$\begin{array}{cc} \mathrm{UTS}\;\left[\mathrm{MPa}\right]= & \;4.1+0.417T_{ext}+0.2556T_{bed}+0.744v\\ & +467h-0.00394\left(T_{ext}\right)v-2.239\left(T_{ext}\right)h\\ & -7.38vh+0.0314\left(T_{ext}\right)vh \end{array}$$

However, in order to have a more definitive test for the performance of the prediction model, a validation dataset was created by testing specimens printed with parameter combinations that had not been used before to calculate the prediction model (as mentioned in Section 2.3.1). The average UTS results for each parameter combination in the validation dataset are presented in Table 4.

**Figure 7:**
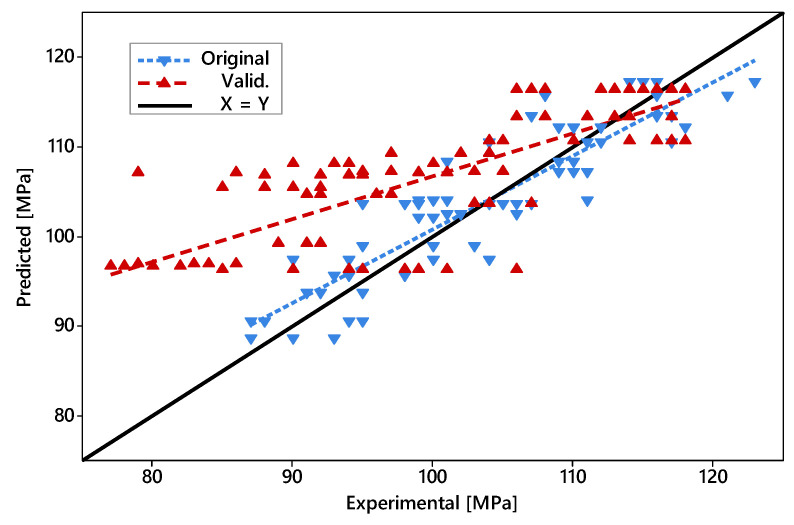
Scatterplot of predicted UTS (based on the DoE model) against the experimental UTS. “Original” denotes the points originally used to calculate the prediction model; “Valid.” denotes the validation points. Dashed lines were fitted based on each set.

By plotting the Experimental UTS values of all data points against their respective predicted UTS values (based on Equation (2)), it is fairly clear that the model performed poorly, predicting UTS values within the lower ranges of the spectrum comprising all observed outcomes (Figure 7). This is evidenced by the large difference between the fitted lines based on data points from both datasets. However, the same fitted lines converged towards higher UTS values (110 to 120 MPa) in a point next to the X = Y line, meaning that the model tends to perform better when high UTS values are expected. Thus, although not ideal, such a model can still be useful to predict a parameter combination resulting in the highest possible UTS within the evaluated ranges.

By maximizing the function given by Equation (2) (within the evaluated ranges), the maximum of the function within the evaluated ranges (Table 1) could be located at one of the edges of the hypercube defined by the data points from the original dataset (see Figure 3). This was a direct result of the lack of curvature associated with the model (the curvature had a *p*-value of 0.486, see Table 3). Consequently, according to this prediction model in particular, the relationship between UTS and each main factor was considered linear.

**Figure 8:**
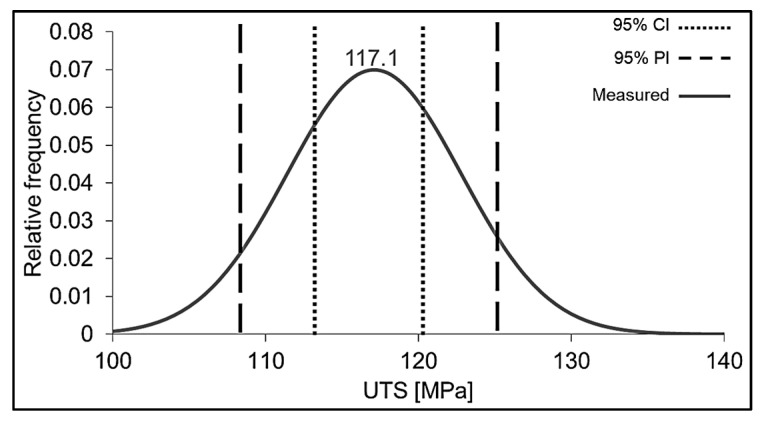
Distribution of UTS values for specimens 3D-printed with the optimal parameter combination, according to the DoE prediction model. Confidence and prediction intervals are delimited according to Table 5.

To verify the calculated solution and its predicted UTS value, eight specimens were produced using the optimized set of parameters (Table 5) and then mechanically tested. The result was an average UTS of 117.1 ± 5.7 MPa, which is close to the predicted one (and therefore within the 95% confidence interval, CI). The 95% prediction interval (PI) contained 90% of the population, a difference that could be considered reasonable. The results from the verification of the optimal solution are graphically visualized in Figure 8.

#### 3.1.2. Prediction Model Based on Polynomial Regression Applying ML Principles

**Figure 9:**
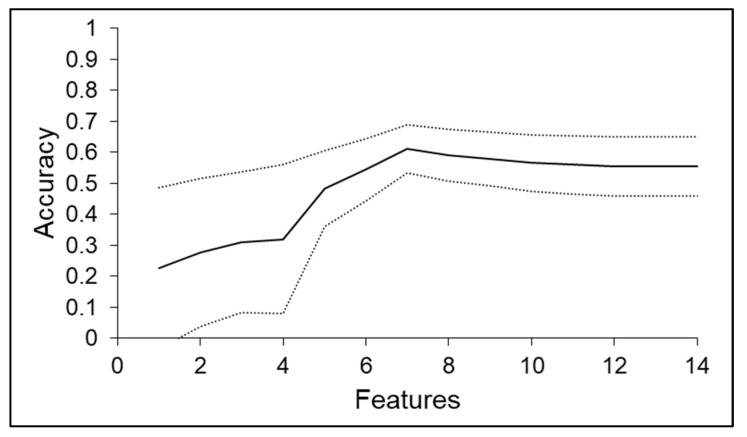
Relationship between cross validation accuracy score and total number of features present in the intermediate ML-based model. The black line corresponds to the mean CV score, whereas the dashed lines represent an interval of plus or minus one standard deviation.

According to the ML-based algorithm deployed in the present study, the evolution of the CV score (or model accuracy) with the removal of features using the RFE method was crucial to determine the final model. The CV score/model features relationship can be seen in Figure 9. The mean CV score peaks at seven features (out of a total of 14) with a value of 0.61 ± 0.08. According to the literature, algorithms using RFE tend to perform better as the number of informative features retained by the model increases until a maximum is reached. In many cases a plateau is reached from this point onwards, as reported in the literature [41,42,45,46,47,48].

**Figure 10:**
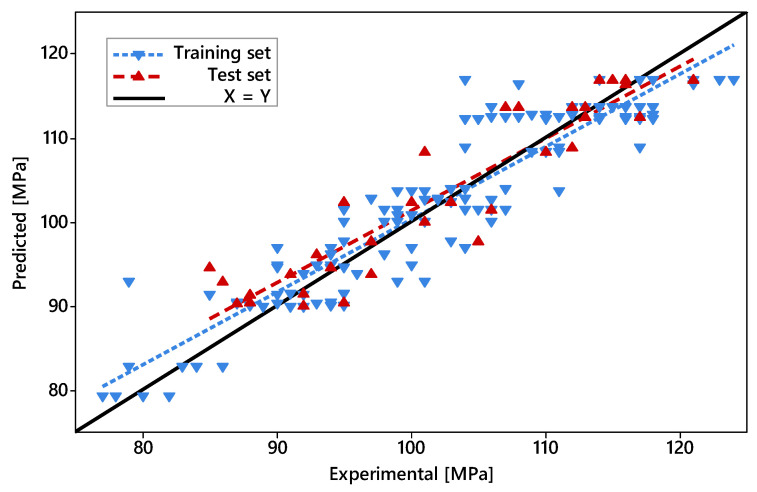
Scatterplot of predicted UTS (according to the ML-based model) against the experimental UTS. Dashed lines were fitted based on each set.

With the number of features fixed at seven, the final prediction model based on ML was established, described by Equation (3). The summary of the model metrics is presented in Table 6. According to the metrics, the coefficients of determination calculated for both training and test sets were similar, which suggests that this model could generalize reasonably well to new presented data within the evaluated parameter windows. Compared to the DoE, the ML-based model was more accurate, especially in observations where lower UTS values (90 to 110 MPa) were expected (see Figure 10). It stands to reason that the quadratic terms obtained from the ML-based algorithm enhanced the chances of the model perceiving non-linear relationships—features that were lacking in the more simplistic 2k full factorial DoE model—ultimately improving its reliability. However, both models converged at higher predicted UTS values, and as such, culminated in similar optimized solutions (117.3 and 117.4 MPa for ML-based and DoE, respectively), although with slightly different parameter combinations (*T_bed_* = 112 °C and *T_bed_* = 120 °C for ML-based and DoE, respectively, with all other parameters values kept as presented by Table 5). The C-opt parameter combination applied on the ML-based model results in a predicted UTS of 114.5 MPa, which corresponds to a 2.2% difference to the actual average (see Figure 8). Although, arguably, such a difference can hardly be considered significant, it demonstrates that in the present case the DoE model fared better in predicting an optimal solution, despite being the less accurate model overall.

(3) $$\begin{array}{cc} \mathrm{UST}\;\left[\mathrm{MPa}\right]= & \;-361.599-0.159T_{ext}+10.942T_{bed}\\ & -2.381v-501.43h+0.00531\left(T_{ext}\right)v\\ & -0.0487T_{bed}{}^{2}+0.00816v^{2}+708.6h² \end{array}$$

As a final remark concerning the process optimization, it should be emphasized that the parameter windows evaluated during the production of both prediction models were recommended by the filament supplier beforehand. Therefore, although significant differences in UTS could still be observed across the different conditions, the influence of each process parameter on the response was attenuated by this fact. By broadening the parameter windows, one could expect that considerably worse conditions would also be present in the datasets. In turn, this would accentuate the influence of parameter selection on the observed response variance, possibly improving model metrics such as R² and RMSE consequently. In spite of this, process parameters and their interactions did play significant roles in the tensile test outcome. The next sections are dedicated to addressing these roles, both quantitatively and qualitatively, comparing model results to microstructure and temperature evolution during the manufacturing process.

### 3.2. Material Behavior under Load: General Aspects

**Figure 11:**
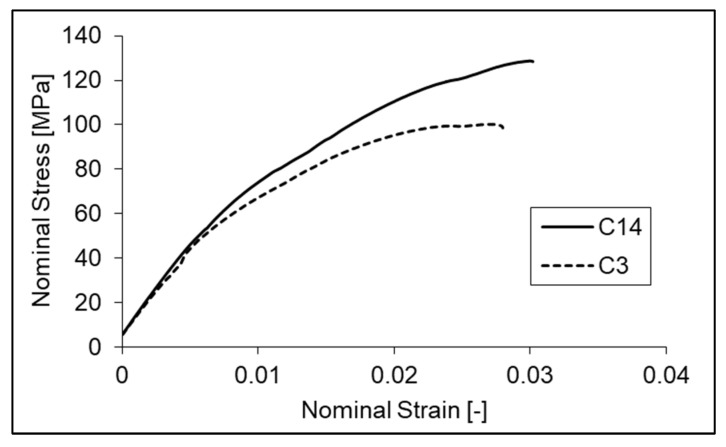
Representative nominal stress vs. nominal strain curves from tensile testing of 3D-printed specimens.

Before the distinctions between different conditions are highlighted, their commonalities must be pointed out. In general terms, the 3D-printed specimens showed limited plastic deformation during the tensile test. Figure 11 shows nominal stress vs. nominal strain curves from exemplars of the best and worst conditions of the original dataset (i.e., the one used to produce the DoE model, Table 2). Values aside, the shapes of these curves are representative of all the tensile tests performed in the present study, regardless of the parameter combination.

**Figure 12:**
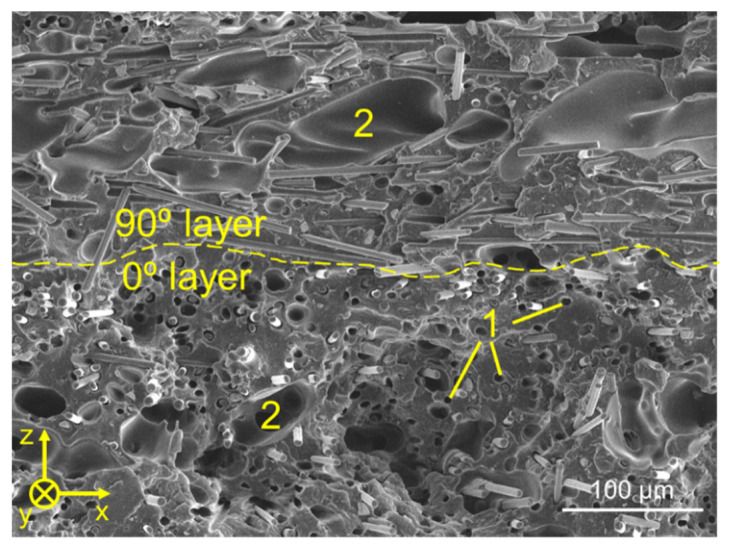
General appearance of fracture surfaces of 3D-printed specimens after tensile testing, with the load direction parallel to the y axis. The dashed line represents the transition between different layers; their orientation angle alludes to their respective printing directions, being relative to the y axis. Cavities caused by fiber pull-out and voids originated from filament fabrication are represented by 1 and 2, respectively.

Beyond a common mechanical behavior macroscopically, general aspects could also be observed upon initial microstructural analysis and fractography, which provided further insight into the behavior of the specimens under load. The first common aspect found across all tested specimens was the fiber orientation with respect to the road. On FFF, short fibers tend to maintain the orientation of the deposited roads [49,50,51,52,53,54], which can potentially increase the anisotropy of the printed part depending on the layer stacking sequence. As a result of this preferential fiber orientation, fibers in 0° layers could be observed mostly parallel to the load direction (see y-axis on Figure 12), while fibers in 90° layers were perpendicular to it.

This common aspect can be directly related to the next one, namely the presence of cavities of around 5 μm in diameter across the fracture surface (marked with “2” in Figure 12). These cavities were a direct result of the mechanical test, appearing from the fiber pull-out mechanism taking place under load. Furthermore, they were mostly observed in the 0° layers, which had the fibers aligned parallel to the load direction. In this situation, the crack propagation occurred perpendicularly to the fiber orientation, which is characterized by mechanisms such as fiber debonding followed by pull-out and/or fiber breakage [55].

**Figure 13:**
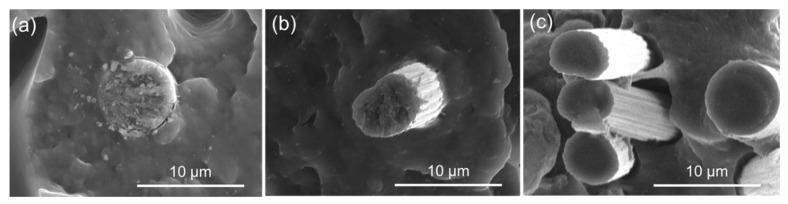
Fracture mechanisms observed on 0° layers. (**a**) Fiber breakage; (**b**) Limited debonding leading to breakage; (**c**) Extensive debonding leading to fiber pull-out.

As cracks propagated across fiber-reinforced regions, three situations could be expected. (a) If there was a strong interaction between fiber and matrix, no fiber debonding took place, resulting instead in its breakage; (b) if the fiber–matrix interactions were weaker, then fiber breakage occurred, preceded by limited debonding; or finally, (c) if fiber–matrix interactions were weak enough, extensive debonding took place until the fiber was pulled out, with no breakage [55]. While all three situations were observed in 0° layers (see Figure 13), (c) was predominant, being responsible for 50.8% of all registered matrix–fiber interaction events (*n* = 457), compared to 34.6% of (b) and 14.7% of (a). Those results indicate a weak interaction between fiber and matrix [44]. The poor interfacial adhesion between CF and polyamide matrices has been addressed by many studies [56,57,58,59,60], being caused by the fact that CF and thermoplastic matrix are mostly inert and unreactive towards each other [59].

**Figure 14:**
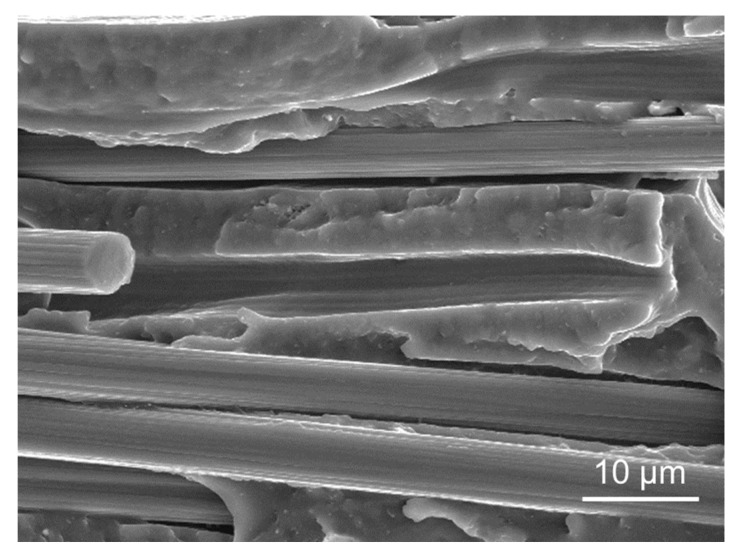
Fiber debonding observed on fracture surfaces of 90° layers.

In 90° layers, crack propagation was parallel to the fiber orientation instead. Since the crack propagation path is more straightforward in this case (which is indicated by a relatively plain fracture surface [55]), this type of fracture is associated with lower energies compared to those observed in the 0° layers. When fiber and matrix interact weakly with each other (which appeared to be the case, as discussed), this type of failure is also characterized by the presence of fiber debonding, which could be observed in 90° layers (see Figure 14). Large gaps between fiber and matrix were identified in all samples, as well as imprints left by detached fibers.

**Figure 15:**
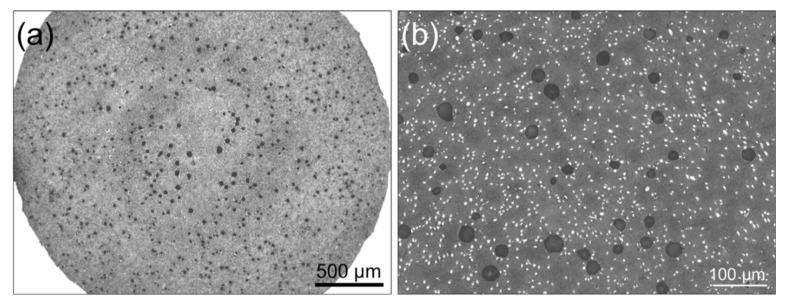
(**a**) Cross section of the filament as received. (**b**) The same cross section in closer detail, where white dots represent carbon fibers and darker regions represent pores that originated in the filament fabrication.

Lastly, another common aspect among all tested specimens was the presence of intra-road voids (see Figure 12, where those pores were labelled as 2). Such voids were already present in the filament (see Figure 15) and are a direct consequence of the poor incorporation of short fibers into the thermoplastic matrix during the filament fabrication process. According to several authors [12,49,50,51,52,61,62], many filaments reinforced with short fibers (such as PA, PEEK, ABS and PLA) are prone to having such porosity due to the relatively poor adhesion between the thermoplastic and the CF surface [61,63,64], as briefly mentioned before. For ABS in particular, Ning et al. [12] reported that this particular type of porosity tends to increase from 0 to 10 wt.% of short fiber reinforcement, followed by a decrease from 10 to 15 wt.%. This variation can be directly correlated with the mechanical performance of the ensuing 3D-printed parts.

### 3.3. Influence of Process Parameters on the Mechanical Performance

**Figure 16:**
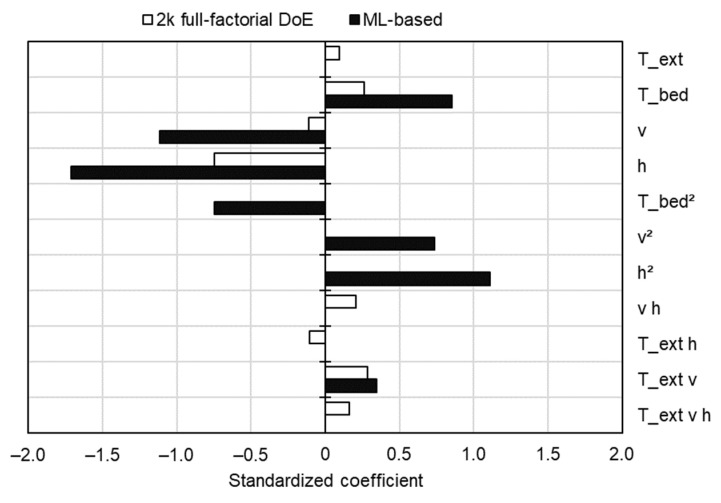
Standardized regression coefficients for each feature present in both the DoE and ML-based models.

Standardized regression coefficients (β_i_*) for each prediction model were calculated according to Equation (4), where *s_xi_* and *s_y_* represent the estimated standard deviation of independent and dependent variables, respectively, and *β_i_* represents the initial coefficients as given by the model equations. This method has been described in the literature [65,66] and allows for a direct comparison of how much each model relies on each feature, as shown in Figure 16.

(4) $$\beta_{i}^{*}=\frac{s_{x}{}_{i}}{s_{y}}\beta_{i}$$

Initially, such a comparison presented noticeable discrepancies. For one, the standardized regression coefficients had much greater absolute values in the ML-based model in comparison with the DoE one. Moreover, as already mentioned, the ML-based model relies heavily on the quadratic terms, whereas the DoE one draws important contributions from two and three-way interactions. However, despite those discrepancies, similarities between the feature contributions arise upon closer inspection. The influence of each feature on the response pointed in the same direction for either model, i.e., both models agreed that *T_bed_* influences the UTS positively, while *h* influences it negatively. Furthermore, although linear terms such as *T_bed_*, *v* and *h* had much greater contributions in the ML-based model, they were balanced out by also large (though smaller) contributions of their respective quadratic terms in the opposite direction. Finally, the interaction with the largest contribution in the DoE model (*T_ext_ v*) is also the only one present in the ML-based model. In the following sections, the causes of the observed effects will be addressed for each individual parameter, based on microstructural analysis prior to and after testing, as well as on temperature measurements performed during the FFF process. Contour plots generated with DOE and ML-based prediction models are available in the Appendix A and Appendix B, respectively.

#### 3.3.1. Influence of Layer Height, *h*

**Figure 17:**
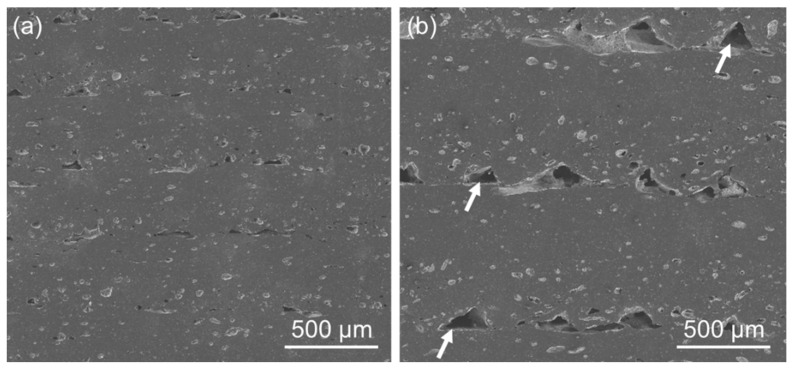
Cross sections of untested specimens printed with a layer height of (**a**) 0.2 mm (C14) and (**b**) 0.4 mm (C6). Gaps between beads seen in (**b**) are marked with arrows. Spots in light grey were voids that were filled with carbon during the sputtering prior to SEM.

Comparisons between models aside, the layer height appeared to be the most influential parameter on the mechanical resistance of the 3D-printed specimens. As shortly mentioned before, the effect on the mean UTS value was negative, meaning that higher *h* values resulted in weaker parts. This observation has also been reported by other authors [67,68,69,70] for PLA. In the present study, a closer look at the cross sections of untested specimens with different layer heights revealed that higher *h* values resulted in more interlayer gaps, see Figure 17.

These discrepancies in porosity can be understood as follows: for all experiments, the road width remained constant at 0.4 mm, with a constant air gap of 0.4 mm (see Table 1). Considering those restraints, a layer height of 0.4 mm resulted in each individual road having enough space to be deposited roughly as a cylinder, resulting in gaps left between the neighboring roads (marked with arrows in Figure 17b). In turn, those gaps resulted in less material in contact with the underlying layer, which consequently decreased interlayer cohesion. Moreover, with abundant inter-road gaps, the effective cross-section area decreased, which consequently would also undermine the mechanical resistance of the 3D-printed part in comparison to a theoretical monolithic one with the same dimensions [67,71]. On the other hand, when *h* was reduced to 0.2 mm, pore formation between layers was found to be reduced. Some authors [72,73] ascribed this pore suppression to a road spreading effect, induced by the proximity of the nozzle to the surface of the previous layer. As a consequence, the contact surface between layers occurred over a greater area, leading to a higher interlayer cohesion and hence better mechanical performance.

**Figure 18:**
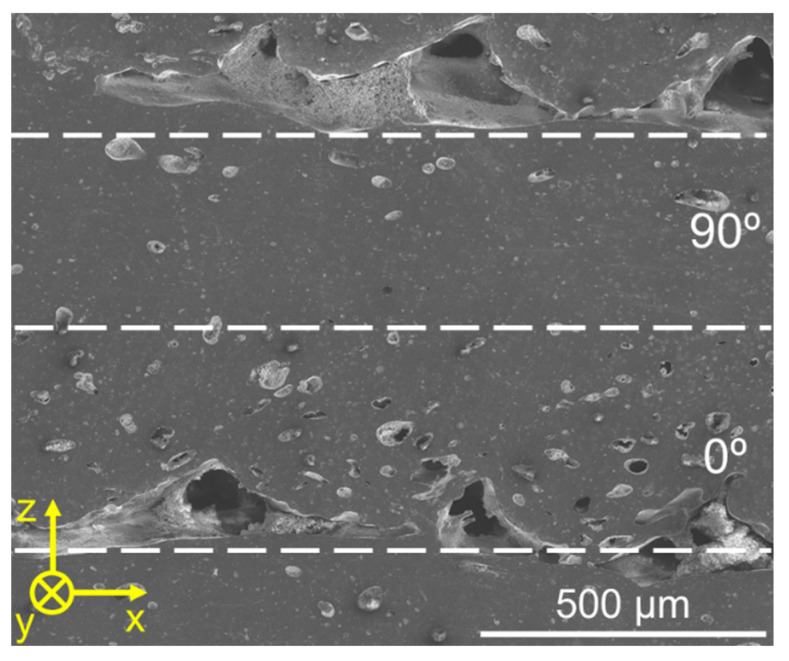
Cross section of C6 before testing. Dashed lines indicate the layer orientation boundaries; 0° layers were printed parallel to the y axis, whereas 90° ones were printed perpendicular to it.

**Figure 19:**
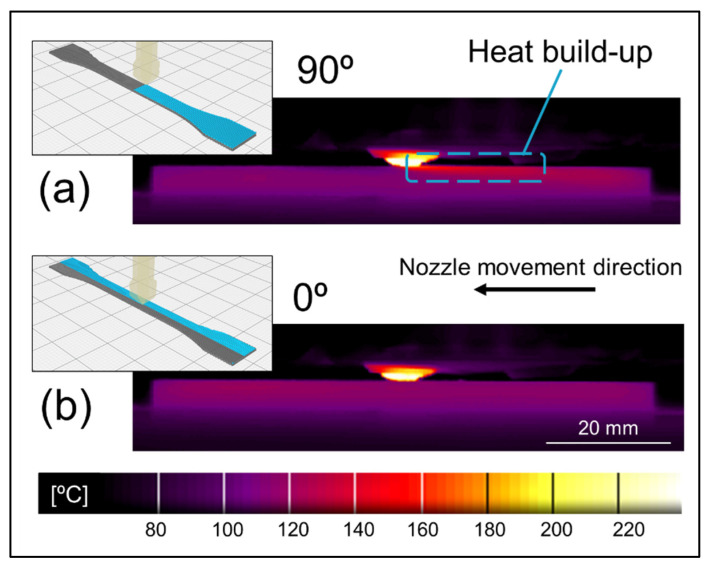
Infrared temperature measurement during printing of (**a**) 90° layers and (**b**) 0° layers. Blue lines represent completed roads from the current layer. Gray lines represent beads from the underlying layer.

Furthermore, the referred inter-road gaps appeared predominantly at the bottom of 0° layers, while the upper areas of those layers were virtually devoid of such gaps, see Figure 18. This observation can be related to the temperature gradients developing during the deposition of different layers, which can be visualized in Figure 19. On the deposition of 90° layers, the shorter paths required to deposit each road (“lateral movement”) led to a heat build-up effect trailing the nozzle, which decreased the cooling rate in those regions. On the other hand, when a 0° layer was deposited, the nozzle traveled across much longer paths (“longitudinal movement”) and consequently the heat build-up was not as prominent. Sun et al. [74] previously reported this effect, also establishing a correlation between the effect itself and the degree of sintering between neighboring roads. According to those authors, specimens printed with only 90° layers displayed a larger neck radius in comparison to the ones printed with only 0°, which could be directly attributed to the higher average temperature achieved by the former during its production. In the present case, where the layer orientation alternated between 90° and 0°, the deposition of the former led to greater sintering between current and previous layers, which in turn suppressed the formation of inter-road gaps.

Nevertheless, it stands to reason that the development of the aforementioned temperature gradients (and the consequent porosity distribution) occurred in the described way only due to the particular shape of the 3D-printed specimens, which in this case were rather elongated, i.e., with a high aspect ratio. If the aspect ratio of the 3D-printed part was smaller (as in a cube-shaped model, for instance), discrepancies in heat build-up across layers with different orientations would tend to be lessened.

#### 3.3.2. Influence of Printing Bed Temperature, *T_bed_*

In either prediction model created in the present study, the bed temperature also had a significant impact in the mechanical performance of printed specimens. As opposed to the layer height *h*, the influence of *T_bed_* on the mean UTS value was positive, meaning that higher temperatures resulted in higher tensile strength values. Such an effect has also been reported elsewhere [72,75,76,77].

**Figure 20:**
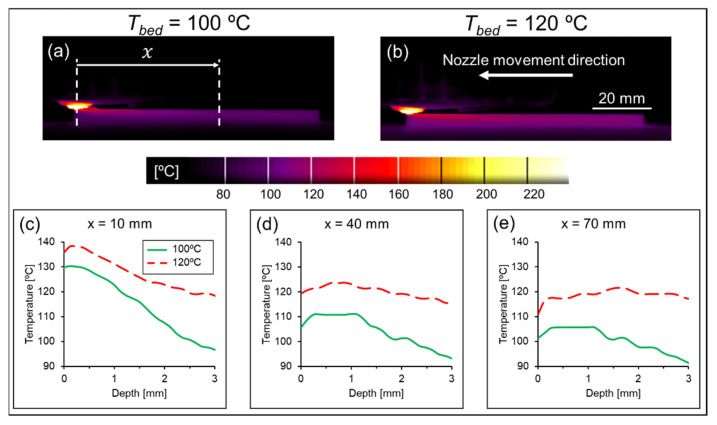
Comparison between the temperature profiles of specimens printed at a bed temperature of (**a**) 100 °C and (**b**) 120 °C. The nozzle moved longitudinally during the measurement. The profiles were measured at (**c**) 10 mm, (**d**) 40 mm and (**e**) 70 mm from the nozzle.

This correlation can be better understood by comparing the actual temperature distribution during the process with different *T_bed_* values. When decreasing *T_bed_* from 120 °C to 100 °C while keeping *T_ext_* constant (280 °C), it could be observed that the whole 3D-printed part became significantly colder, as expected. Moreover, this temperature difference between both conditions increased with the distance from the nozzle, *x*, see Figure 20. Sun et al. [74] argued that printing at a surrounding temperature above *T_g_* induces healing, i.e., the molecular diffusion between neighboring roads. Despite either condition being above *T_g_*, higher temperatures tend to accelerate the intermolecular diffusion [78], leading to a better interlayer cohesion and therefore stronger 3D-printed parts.

**Figure 21:**
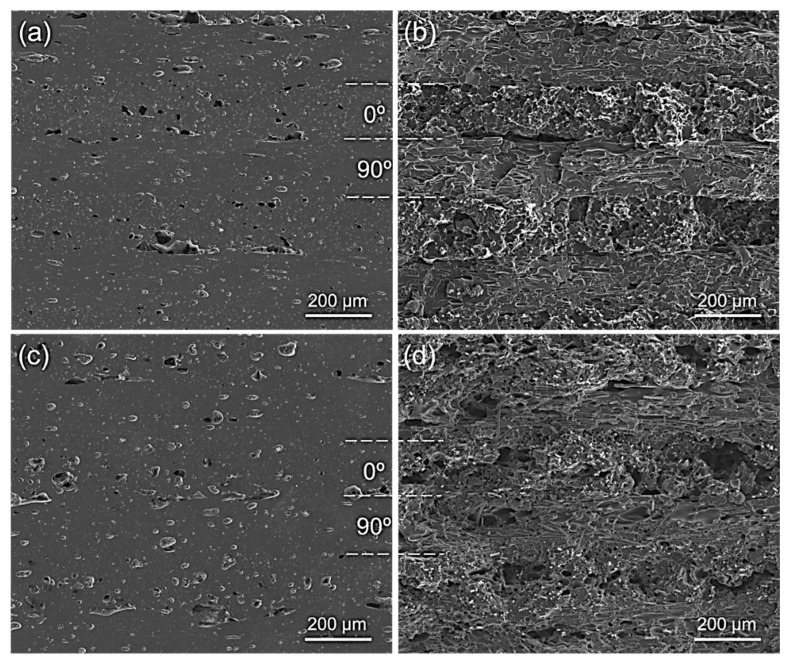
Cross sections prior to mechanical testing and their respective fracture surfaces of specimens printed at different printing bed temperatures. (**a**) 100 °C before mechanical testing (C13); (**b**) 100 °C after mechanical testing; (**c**) 120 °C before mechanical testing (C14); (**d**) 120 °C after mechanical testing.

Aside from mechanical performance (which can also be attributed to other factors such as porosity), the interlayer cohesion can be inferred by observing the distinction between the layers themselves. It is reasonable to expect that this distinction becomes clearer with lower printing bed temperatures, particularly when the microstructure is observed after the fracture [72,74]. In the present case, this layer differentiation could be somewhat observed in C13 (*T_bed_* = 100 °C), especially in contrast to C14 (*T_bed_* = 120 °C), where the layer interfaces were visually less distinct (compare Figure 21b–d).

However, similar to the influence of the part geometry on the development of localized heat build-up (discussed in Section 3.3.1), the relatively low specimen thickness may have boosted the influence of *T_bed_* on the mean of UTS. Since the heat supplied from underneath could influence even the deposition of the highest layers, specimens were mostly homogeneous across the thickness (see Figure 21). For taller parts, however, the surrounding temperature decreases exponentially with each new layer [79], affecting the interlayer cohesion and consequently reducing the influence of *T_bed_* on the overall mechanical performance. To compensate this effect, an enclosed heated chamber can be used [29,79,80,81].

#### 3.3.3. Influence of Extrusion Temperature, *T_ext_*, and Printing Speed, *v*

**Figure 22:**
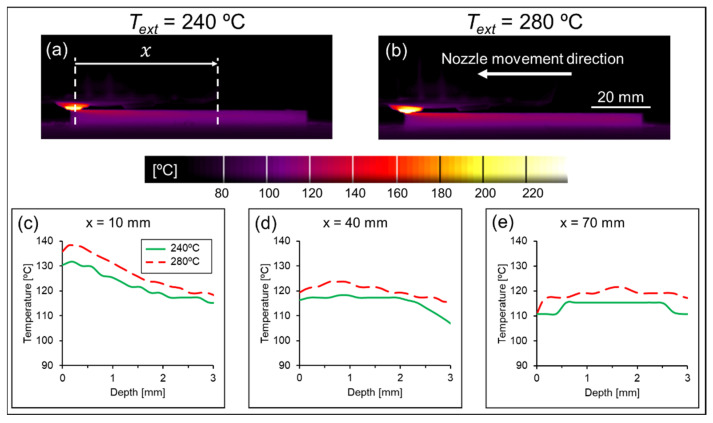
Comparison between the temperature profiles of specimens printed at an extrusion temperature of (**a**) 240 °C and (**b**) 280 °C. The nozzle moved longitudinally during the measurement. The profiles were measured at (**c**) 10 mm, (**d**) 40 mm and (**e**) 70 mm from the nozzle.

The linear terms representing *T_ext_* and *v* did not exert as great of an influence as *T_bed_* and *h* on the responses of either prediction model. On one hand, the DoE model indicated that the contributions of *T_ext_* and *v* to the UTS cannot be considered statistically significant (*p*-values of 0.091 and 0.053, respectively), which was merely enough to bypass the backward elimination steps (α = 0.10 to remove). Particularly for *T_ext_*, the differences in temperature evolution when this parameter was varied from 240 °C to 280 °C were milder (Figure 22) when compared to the differences observed when varying *T_bed_* (Figure 20). On the other hand, while the ML-based model also considered *T_ext_* not relevant for predicting the response (5th feature to be removed by RFE), the influence of *v* was considered significant, even taking into account the opposite effect of *v*^2^ (standardized effects of −1.11 and 0.73, respectively, see Figure 16). Similar to the influence of *h* in either prediction model, the ML-based one considered *v* as having a negative effect on the UTS, i.e., lower speeds tended to increase the response.

**Figure 23:**
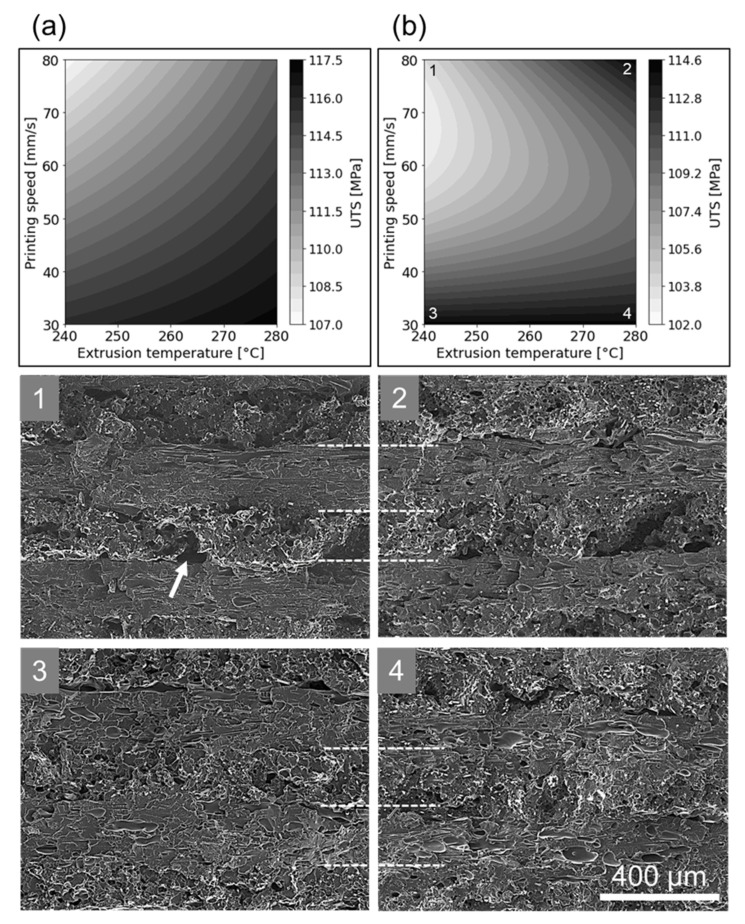
Contour plots for *T_ext_* and *v* based on the (**a**) DoE and (**b**) ML-based prediction models, with *T_bed_* and *h* kept at 120 °C and 0.2 mm, respectively. Fracture surfaces labeled with numerals (**1**–**4**) were obtained from specimens mechanically tested at FFF parameter combinations indicated in (**b**). White dashed lines in the images of fracture surfaces indicate the estimated transition between successive layers. Arrow in 1 indicates an inter-road gap.

However, both process parameters addressed in this section can be better understood as a single factor represented by their interaction, which was considered significant in both prediction models. In qualitative terms, this interaction can be seen as the energy input per time per area (or volume) during the deposition of a given layer, which in other additive manufacturing processes is frequently termed “energy density” [82,83,84,85,86]. The effects of this interaction on the UTS can be visualized by contour plots based on the regression equations, as presented in Figure 23a,b. Since the prediction models themselves present discrepancies, the contour plots appeared different when compared to each other, although showing a similar trend, that is, higher UTS values achieved towards regions with higher *T_ext_* and lower *v*.

However, being the more accurate one overall, the ML-based prediction model could be better related to the observed microstructure, as shown in Figure 23(1–4). On one hand, parameter combinations with low *v* (bottom of the Figure 23b) resulted in printed specimens with similar fracture surfaces, where the transition between layers is of hard identification (Figure 23(3,4)). As discussed previously, this indicates good interlayer cohesion, in turn resulting in better mechanical performance, which was indeed predicted by the contour plot of Figure 23b.

On the other hand, specimens produced with high *v* (top of the Figure 23b) generally presented a clearer visual differentiation between adjoining layers. This differentiation is decreased, however, with an increase in *T_ext_* (Figure 23(2)), becoming somewhat comparable to the specimens printed with lower *v*.

According to the ML-based model, conditions presented by Figure 23(2–4) are expected to yield similar responses. When printing at high *v* and low *T_ext_* (i.e., where the ML-based model would predict a decrease in the UTS, see top left corner of Figure 23b), it could be observed that such a parameter combination not only resulted in the clearest visual interlayer transitions, but even resulted in some inter-road gaps as well (see arrow in Figure 23(1)). Those gaps were similar to the ones observed in experiments with a higher *h* (see Figure 17b).

A correlation with the latter observation can be found in the literature. In a study modeling the influence of process parameters on the interlayer cohesion of 3D-printed ABS specimens, Sun et al. [74] proposed a differentiation between the terms “healing” and “sintering”. Healing would represent the intermolecular diffusion from one layer to the other, whereas the sintering would be related to necking formation driven by surface tension. The authors observed that sintering only occurred shortly after the deposition, while the local temperature was still above the critical sintering temperature. During this time, necking occurred, reducing the gaps between adjacent roads.

Based on those findings, in the present case, the time interval for sintering (and consequently the neck radius between adjacent roads) could be expanded either by increasing the extrusion temperature *T_ext_* or reducing the printing speed *v*. By printing fast and using lower extrusion temperatures, no significant sintering effect could occur, which resulted in the emergence of inter-road gaps that ultimately affected the mechanical performance.

## 4. Conclusions

In this study, a commercial-grade polyamide filament reinforced with short carbon fibers was used to 3D-print tensile test specimens by fused-filament fabrication. Extrusion and printing bed temperature, as well as layer height and printing speed were varied. Upon testing, specimens showed limited plastic deformation, regardless of the parameter combination. Extensive fiber pull-out and fiber debonding could be observed, as well as voids that could already be identified in the as-received filament. Those voids were a direct result of the fiber incorporation during the filament fabrication and have also been reported by other authors with different short fiber reinforced filaments.

Two models for predicting the mechanical resistance of 3D-printed specimens were proposed, taking into account the evaluated process parameters. The models were constructed with two different methodologies: one based on a 2k full factorial design of experiments and another based on a multiple polynomial regression adopting machine learning principles. It was concluded that when submitted to new observations, the ML-based model predicted the response with higher accuracy. However, the DoE fared slightly better at predicting observations where higher response values were expected, including the optimal solution, which reached an experimental ultimate tensile strength value of 117.1 ± 5.7 MPa.

There was an important correlation between the process parameters and the response. Layer height and printing bed temperature were considered the most influential parameters, while extrusion temperature and printing speed had a lower influence on the outcome. The general influence of the parameters on the response was correlated qualitatively with the degree of interlayer cohesion, which in turn affected the mechanical performance of the 3D-printed specimens. Situations where sintering was hindered (i.e., higher layer heights, colder extrusion and faster printing) resulted in inter-road porosity due to the limited neck growth between adjacent roads, which ultimately undermined the resistance of the specimens. The printing bed temperature was responsible for dictating the overall temperature of the specimens during the process, and as such influenced the intermolecular diffusion between successive layers. This was indicated by the degree of layer differentiation that could be observed especially after fracture, which was decreased with higher printing bed temperatures.

## Figures and Tables

**Table 1:** Evaluated ranges of process parameters.

Parameter	Value
Extrusion temperature (*T_ext_*)	240–280 °C
Printing bed temperature (*T_bed_*)	100–120 °C
Printing speed (*v*)	30–80 mm/s
Layer height (*h*)	0.2–0.4 mm
Nozzle diameter	0.6 mm (constant)
Bead width	0.4 mm (constant)
Air gap	0.4 mm (constant)

**Table 2:** Average UTS values for each condition within the DoE.

Condition	*v* [mm/s]	*T_bed_* [°C]	*T_ext_* [°C]	*h* [mm]	UTS [MPa]
C1	30	100	240	0.4	97.0 ± 5.4
C2	30	120	240	0.4	102.8 ± 1.9
C3	80	100	240	0.4	91.0 ± 3.5
C4	80	120	240	0.4	95.0 ± 2.2
C5	30	100	280	0.4	89.2 ± 2.5
C6	30	120	280	0.4	92.7 ± 1.7
C7	80	100	280	0.4	100.2 ± 3.3
C8	80	120	280	0.4	102.8 ± 4.8
C9	30	100	240	0.2	111.0 ± 4.6
C10	30	120	240	0.2	115.0 ± 5.4
C11	80	100	240	0.2	99.3 ± 0.5
C12	80	120	240	0.2	110.0 ± 0.8
C13	30	100	280	0.2	112.3 ± 3.5
C14	30	120	280	0.2	117.0 ± 3.5
C15	80	100	280	0.2	107.5 ± 3.8
C16	80	120	280	0.2	114.2 ± 4.2
C17	55	110	260	0.3	102.5 ± 4.2

**Table 3:** Summary of prediction model based on DoE.

S [MPa]	R²	R² (Adjusted)	R² (Predicted)	Curvature (*p*-Value)	Lack-of-Fit (*p*-Value)
4.13	0.820	0.795	0.761	0.486	0.757

**Table 4:** Average UTS values for each condition used to validate the prediction model based on DOE.

Condition	*v* [mm/s]	*T_bed_* [°C]	*T_ext_* [°C]	*h* [mm]	UTS [MPa]
E1	75	106	280	0.32	100.0 ± 9.5
E2	30	120	259	0.2	112.7 ± 3.0
E3	50	100	252	0.2	94.3 ± 4.2
E4	40	102	264	0.24	100.0 ± 4.8
E5	30	104	268	0.36	91.0 ± 4.5
E6	55	110	260	0.2	112.8 ± 5.7
E7	35	118	260	0.32	94.0 ± 2.9
E8	45	116	256	0.28	91.3 ± 10.5
E9	55	120	248	0.28	92.3 ± 3.1
E10	65	112	276	0.24	101.3 ± 3.0
E11	60	118	244	0.28	88.8 ± 3.0
E12	55	110	260	0.4	99.9 ± 3.2
E13	60	114	244	0.4	83.0 ± 2.9
E14	80	108	272	0.4	90.8 ± 1.2
E15	70	110	240	0.36	79.3 ± 2.2

**Table 5:** Optimal parameter combination and predicted maximum UTS based on the DoE prediction model.

Condition	*v* [mm/s]	*T_bed_* [°C]	*T_ext_* [°C]	*h* [mm]	Predicted *UTS* [MPa]	95% CI [MPa]	95% PI [MPa]
C-opt	30	120	280	0.2	117.4	(114.3, 120.6)	(108.2, 126.7)

**Table 6:** Summary of prediction model summary based on ML.

S (Train) [MPa]	S (Test) [MPa]	R² (Train)	R² (Test)	CV Accuracy
6.16	5.88	0.682	0.688	0.612 ± 0.078

**Figure A1:**
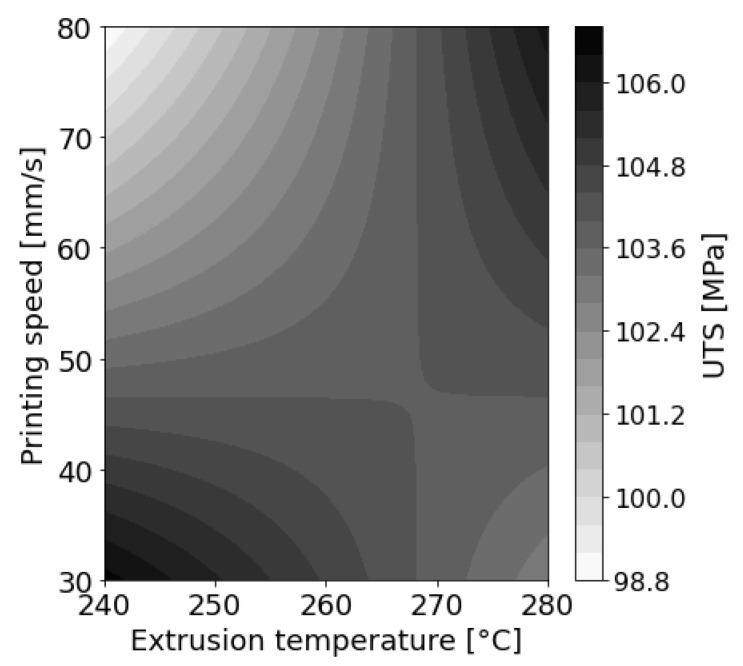
Contour plot for *T_ext_* and *v* based on DoE prediction model.

**Figure A2:**
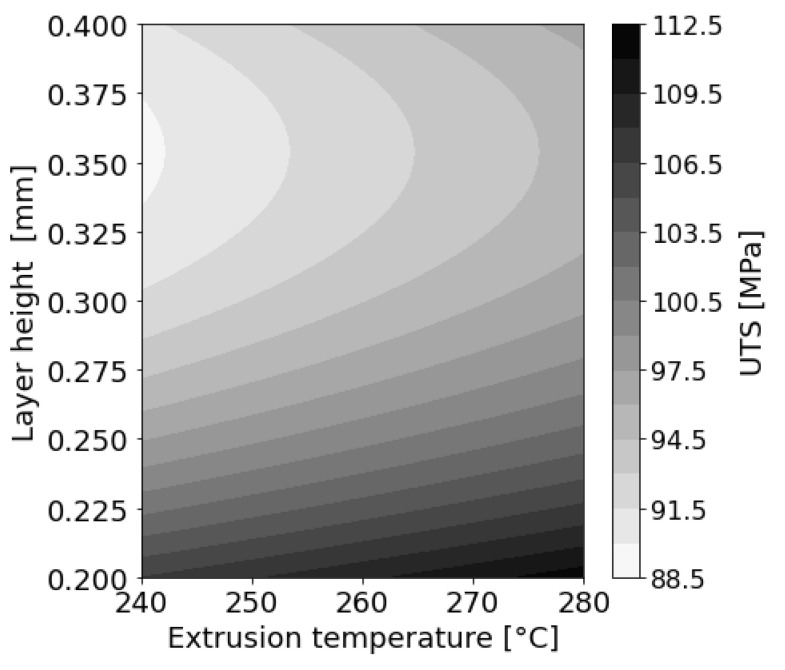
Contour plot for *T_ext_* and *h* based on DoE prediction model.

**Figure A3:**
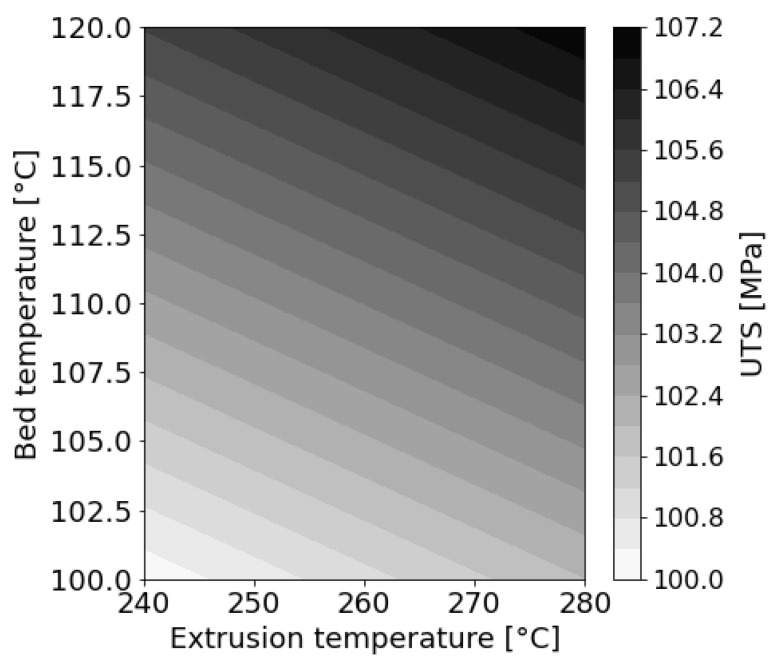
Contour plot for *T_ext_* and *T_bed_* based on DoE prediction model.

**Figure A4:**
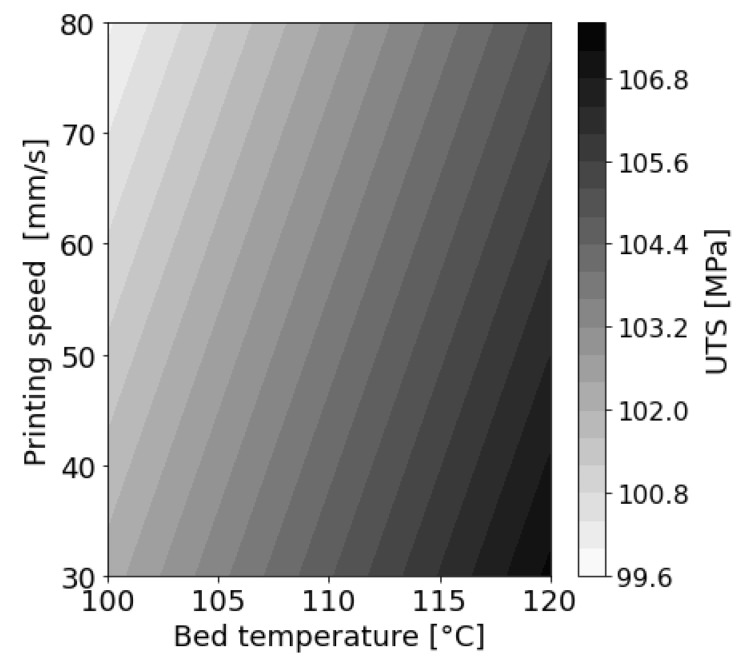
Contour plot for *T_bed_* and *v* based on DoE prediction model.

**Figure A5:**
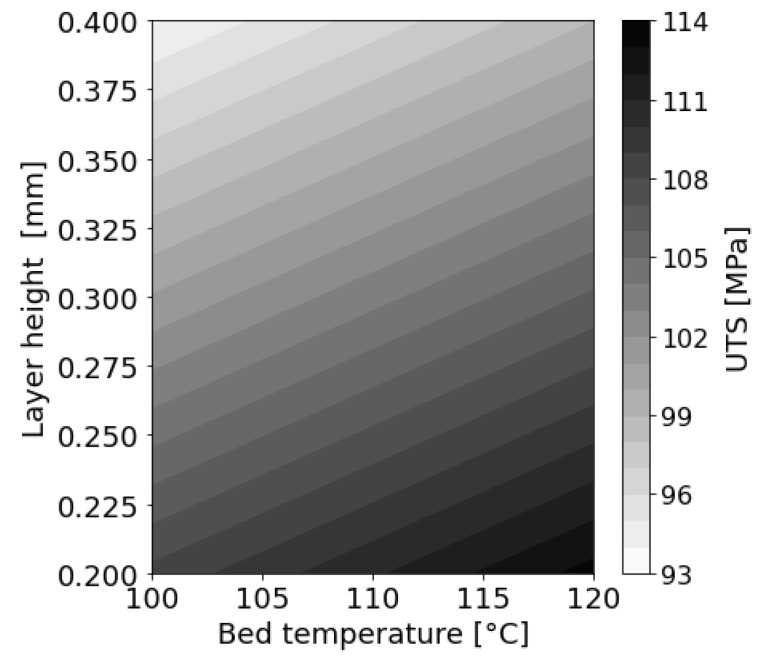
Contour plot for *T_bed_* and *h* based on DoE prediction model.

**Figure A6:**
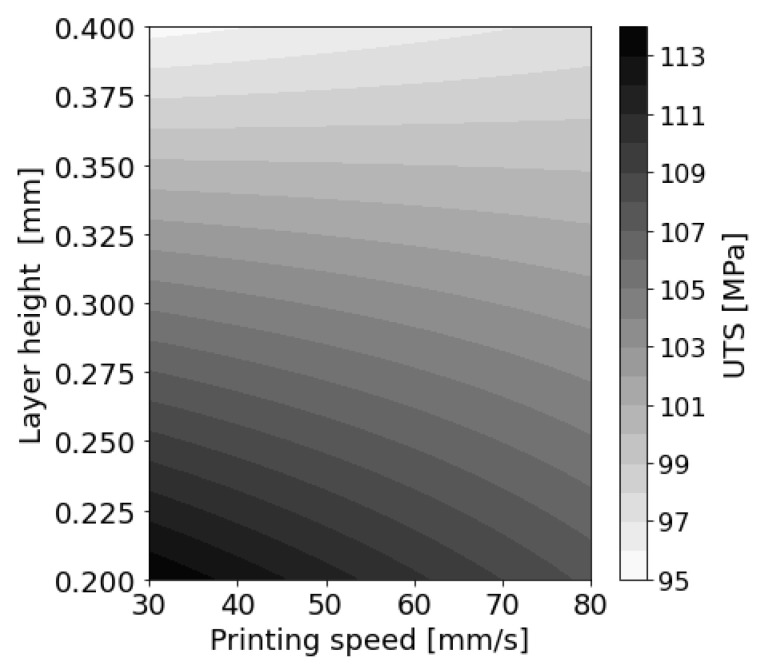
Contour plot for *v* and *h* based on DoE prediction model.

**Figure A7:**
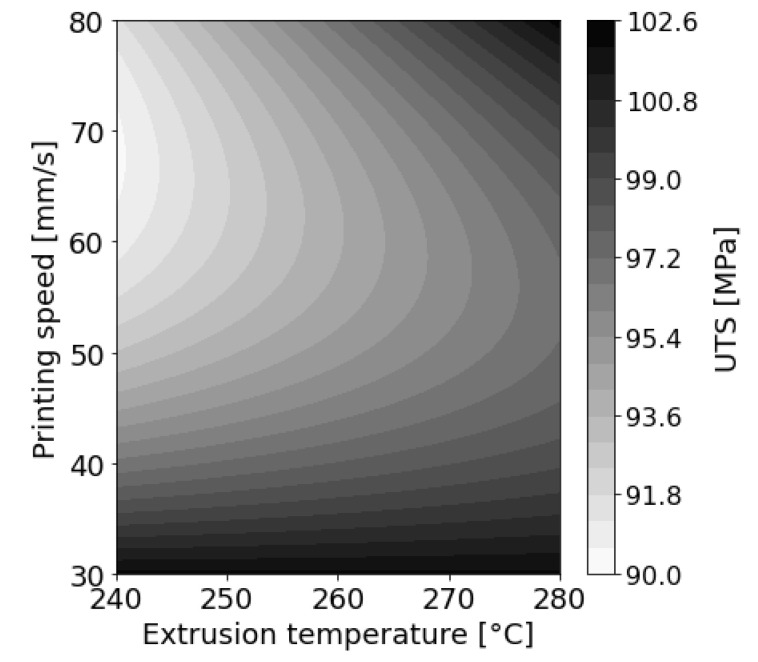
Contour plot for *T_ext_* and *v* according to the ML-based prediction model.

**Figure A8:**
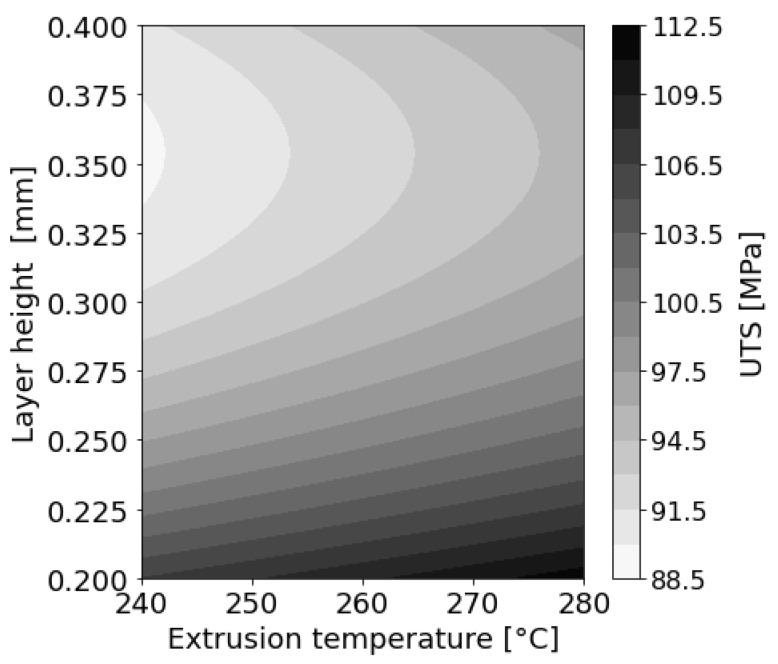
Contour plot for *T_ext_* and *h* according to the ML-based prediction model.

**Figure A9:**
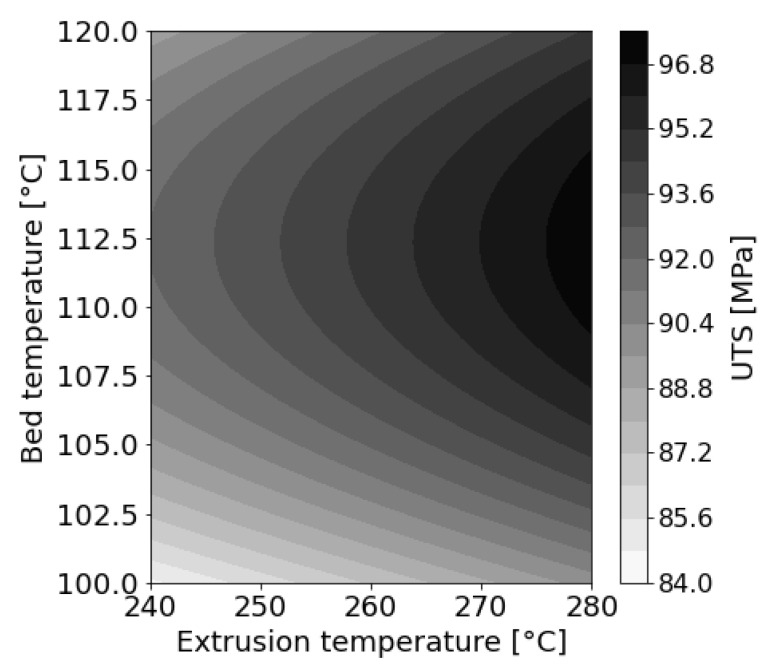
Contour plot for *T_ext_* and *T_bed_* according to the ML-based prediction model.

**Figure A10:**
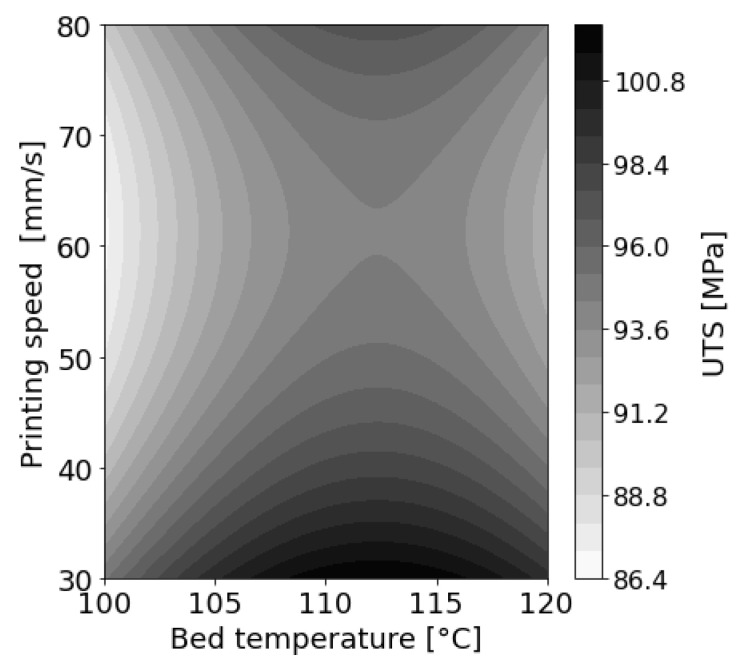
Contour plot for *T_bed_* and *v* according to the ML-based prediction model.

**Figure A11:**
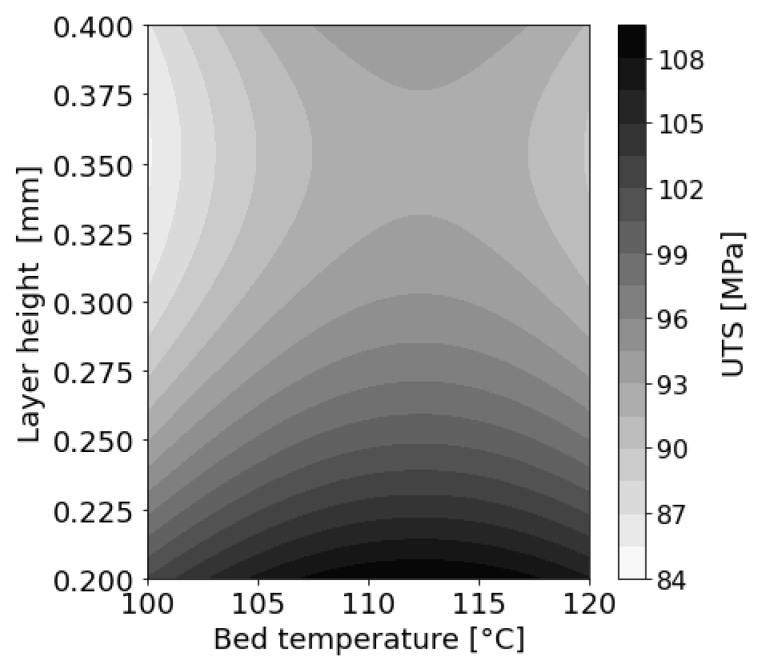
Contour plot for *T_bed_* and *h* according to the ML-based prediction model.

**Figure A12:**
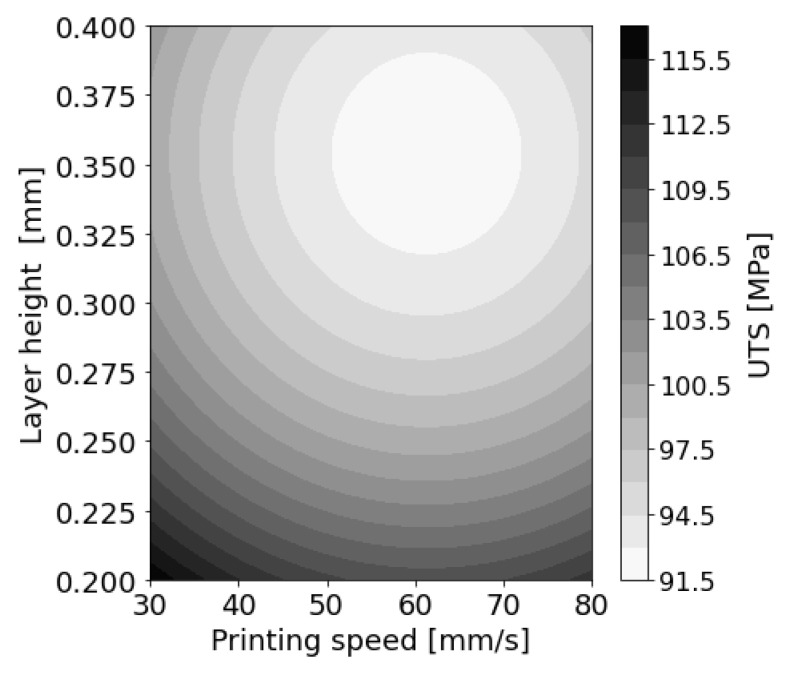
Contour plot for *v* and *h* according to the ML-based prediction model.

## Data Availability

The data presented in this study are available on request from the corresponding author. The data are not publicly available due to it being part of an ongoing project.

## Appendix A

Contour plots based on DoE prediction model. Constant parameters were kept at: *T_ext_* = 260 °C; *T_bed_* = 110 °C; *v* = 55 mm/s; *h* = 0.3 mm.

## Appendix B

Contour plots according to the ML-based prediction model. Constant parameters were kept at: *T_ext_* = 260 °C; *T_bed_* = 110 °C; *v* = 55 mm/s; *h* = 0.3 mm.
